# Association between number of comorbid medical conditions and depression among individuals with diabetes; race and ethnic variations

**DOI:** 10.1186/s40200-015-0171-0

**Published:** 2015-07-07

**Authors:** Maryam Moghani Lankarani, Shervin Assari

**Affiliations:** Department of Psychiatry, School of Medicine, University of Michigan, 4250 Plymouth Rd., Ann Arbor, MI 48109-2700 USA; Center for Research on Ethnicity, Culture, and Health (CRECH), School of Public Health, University of Michigan, 1415 Washington Heights, Ann Arbor, MI 48109-2029 USA

## Abstract

**Background:**

Medical and psychiatric comorbidities are commonly comorbid with diabetes. Race and ethnicity may, however, modify the link between medical and psychiatric comorbidities in individuals with diabetes. In this study we compared Non-Hispanic Whites, African Americans, and Caribbean Blacks with diabetes for the association between number of comorbid medical conditions and lifetime and 12-month major depressive disorder (MDD) in individuals with diabetes.

**Methods:**

Data came from the National Survey of American Life (NSAL), 2001–2003. We included 603 patients with diabetes (75 non-Hispanic Whites, 396 African Americans, and 131 Caribbean Blacks). Number of comorbid medical conditions was the independent variable, lifetime and 12-month MDD were dependent variables, and age, gender, education, marital status, employment, and body mass index were covariates. Race- and ethnic- specific logistic regressions were used to determine race and ethnic differences in the associations between number of chronic medical conditions and lifetime and 12-month MDD, while the effect of all covariates were controlled.

**Results:**

Number of chronic medical conditions was positively associated with lifetime MDD among non-Hispanic Whites (OR = 1.719, 95 % CI = 1.018 – 2.902) and African Americans (OR = 1.235, 95 % CI = 1.056– 1.445) but not Caribbean Blacks (*P* > .05). Number of chronic medical conditions was also associated with 12-month MDD among non-Hispanic Whites (OR = 1.757, 95 % CI = 1.119 – 2.759) and African Americans (OR = 1.381, 95 % CI = 1.175 - 1.623) but not Caribbean Blacks (*P* > .05).

**Conclusions:**

This study shows race- and ethnic- differences in the association between number of medical comorbidities and MDD among patients with diabetes. These findings invite researchers to study the mechanisms behind race- and ethnic- differences in vulnerability and resilience to the mental health effects of chronic medical conditions.

## Introduction

With a trend which is increasing in many countries [1], diabetes currently affects about 350 million people worldwide [2]. With 465 billion dollars direct medical cost, diabetes is responsible for about 10 % of total healthcare expenditures in the United States [1].

Possibly due to its complications [3, 4] and also its effects on activities of daily living and well-being and social life [5–9], individuals with diabetes are at higher risk of depression [10, 11]. The prevalence rate of depression is three-times higher in people with type 1 diabetes and nearly twice as high in people with type 2 diabetes compared to controls [12]. Up to 75 % of adults with diabetes have at least one comorbid medical conditions [13]. High rates of depression and comorbid medical conditions are an essential aspect of diabetes, emphasizing the need for collaborative care for patients with diabetes [14].

Screening, diagnosis, and treatment of comorbid medical and psychiatric conditions - defined as coexisting chronic diseases [11]- should be considered as an essential part of patient care, quality assurance, and evaluation of response to treatment [15]. As most patients with index medical diseases such as diabetes also suffer from comorbid health conditions [15], studying the influence of comorbid conditions on health outcomes helps health care providers to better plan the follow up of patients’ health [16, 17].

Number of chronic medical conditions is one of the commonly used measures of comorbidity in epidemiological and community-based studies [18–21]. In this approach, comorbidity is measured based on number, not type, of medical conditions [22–28]. Studies have shown that number of comorbid conditions is associated with low well-being [29–33], functional status, health related quality of life [34], and higher disability [34] and mortality [35]. Patients with diabetes and comorbid conditions are at higher risk of insulin resistance [13, 36]. Unfortunately, the existing literature on the link between co-morbidity and health and well-being [37–51] does not provide information on this association among patients with diabetes.

Very few studies have tested if race and ethnicity interfere with the effects of medical comorbidities on the mental health of individuals with diabetes or other index diseases. Race and ethnicity determine social class and life experiences, personalities, identities, values, exposures, resources and assets. In this view, even if separate effects of risk and resilience factors are similar across groups, their additive and multiplicative effects may be different across subgroups [52–60]. This can be in part due to differential overlap of risk and protective factors, or different distribution of confounders and mediators. Race and ethnicity shape human’s identity, life experience, values, social power, and access to individual and environmental assets and resources that in turn determine distribution, vulnerability, and resilience of individuals to risk and protective factors [18, 56]. In this view, race and ethnicity operate as contextual factors that modify resilience and vulnerability to the separate, additive, and multiplicative effects of risk and protective factors.

As race and ethnicity have shown to moderate the link between medical and psychiatric conditions [18, 52–60] and in response to the gap of knowledge on race and ethnic differences in the associations between medical and psychiatric comorbidities among patients with diabetes, the current study compared non-Hispanic Whites, African Americans, and Caribbean Blacks with diabetes for the association between number of medical comorbidities and lifetime and 12-month major depressive disorder (MDD).

## Methods

The current study used a cross sectional design. We borrowed data from the National Survey of American Life (NSAL), 2001–2003. The NSAL has been conducted as a part of the Collaborative Psychiatric Epidemiology Surveys (CPES). The study was funded by the National Institute of Mental Health (NIMH).

### Ethics

The study protocol received approval by the Institutional Review Board of the University of Michigan. Participants received monetary compensation. Data was kept fully confidential, and all data were collected, restored, and analyzed in an anonymous fashion. All participants provided written consent.

### Participants

The NSAL methodology, including sampling, process, and interviewer training, has been described elsewhere [61–63]. The NSAL used a national household probability sample of adults (18 years and older). African Americans were residents of large cities, other urban areas, or rural areas. Caribbean Blacks were sampled from large cities.

### Race and ethnicity

African-American individuals were identified as Blacks who did not identify any ancestral tie in the Caribbean. Caribbean Blacks were composed of Blacks who self-identified as being of Caribbean ancestry. The non-Hispanic White population included all Caucasian adults except for those who reported Hispanic ancestry [64].

### Enrollment to the NSAL

The NSAL survey population included US adults (age 18 and older) who were African Americans, Caribbean Blacks or Whites and resided in households located in the coterminous 48 states. The NSAL sample was restricted to adults who were able to complete the interview in English. Institutionalized individuals (e.g. including those in prisons, jails, nursing homes, and long-term medical or dependent care setting) were excluded [64].

### Sampling

The NSAL applied a multi-stage sampling design. The ‘core’ sample for the NSAL was a national area probability sample from which African Americans and Whites were sampled. The sampling also included a special supplemental sample of households in areas of high Caribbean Black residential density. The design of the NSAL Core sample closely resembled the National Survey of Black Americans, 1979–80, which was designed to be optimal for drawing the African-American sample. The NSAL national area probability sample was selected independently of other CPES samples [64].

### Interview

All interviews were conducted in English. For 86 % of the respondents, data was collected through face-to-face computer-assisted interviews. Telephone interviews were used for collection of data of the remaining 14 % of the participants. Interviews lasted 140 min on average. Response rate was 72.3 % overall. The response rate was 70.7 % for African Americans, 77.7 % for Caribbean Blacks, and 69.7 % for non-Hispanic Whites.

### Measures

#### Socio-demographic characteristics

Demographic (age and gender) and socio-economic (marital status, and geographic region of the country) characteristics were control variables in this study. Marital status was operationalized as a three level categorical variable (Divorced/Separated/Widowed, Never Married, and Married) while country of origin was a four level variable (Northeast, Midwest, South, and West).

### Number of comorbid medical comorbidities

Number of comorbid medical conditions was measured using self-reported history of doctor-diagnosed chronic medical conditions, from 13 medical conditions that could occur in addition to diabetes. Respondents were asked about the following conditions: Arthritis/rheumatism, peptic ulcers, cancer, hypertension, chronic liver disease, chronic kidney disease, stroke, asthma, other chronic lung diseases, atherosclerosis, sickle cell disease, heart disease and glaucoma. Self-reported history of doctor-diagnosed chronic medical conditions has been shown to be accurate [65].

### Outcomes

A modified version of the World Mental Health (WHO) Composite International Diagnostic Interview (CIDI) was used to measure lifetime and 12-month MDD. The CIDI is a fully structured diagnostic interview that is designed to measure a wide range of *DSM-IV* based non-psychotic mental disorders. The CIDI has been used in the World Mental Health project [66]. The CIDI is used by trained lay interviewers to generate diagnoses of lifetime and recent DSM-IV-TR / ICD-10 disorders [67]. Clinical reappraisal studies have documented high concordance of CIDI diagnoses with diagnoses made by psychiatrists [68, 69]. Investigation of area under the receiver operating characteristic curve (AUC) has found excellent concordance between CIDI and the Structured Clinical Interview for DSM-IV diagnoses of MDD. Additionally, the prevalence differences between CIDI and Structured Clinical Interview for DSM-IV (SCID) are non-significant at the optimal CIDI diagnostic thresholds. Thus, CIDI operating characteristics are equivalent for MDE to those of the best alternative screening scales [70]. CIDI is also known to provide valid findings for Blacks and their ethnic groups [71–73].

### Statistical analysis

To consider the NSAL’s multi-stage sample design, we used Stata 13.0 for data analysis. For all our analyses, we applied weights due to strata, clusters, and non-response. As a result, results are nationally representative. We used sub-population survey command for all our analyses. Race/ethnic- specific logistic regressions were applied for data analysis. We used number of comorbid medical conditions as the independent variable, demographic and socio-demographics as controls, and lifetime and 12-month MDD as outcomes. Adjusted Odds Ratios (OR) and 95 % Confidence Intervals (CI) were reported. *P*-values less than 0.05 were considered statistically significant.

## Results

Most NSAL participants in all ethnic groups were between 30 and 44 years of age. Almost half of NSAL participants in each ethnic group were women. While most non-Hispanic Whites and African Americans were born inside the US, most Caribbean Blacks were born outside the US. Although most non-Hispanic Whites and African Americans lived in the South, the Northeast represented most Caribbean Blacks (Table 1).

**Table 1:** Demographic and Socio-economic Description of the NSAL participants based on race and ethnicity^b^

	Race/ethnic subgroups			
Demographic data	African American	Caribbean Black	White	Total
	n (%)	n (%)	n (%)	n (%)
Gender				
Men	1271 (44.03)	643 (50.87)	372 (47.26)	2286 (45.87)
Women	2299 (55.97)	978 (49.13)	519 (52.74)	3796 (54.13)
Marital Status				
Married	960 (32.91)	559 (37.56)	383 (47.36)	1902 (40.25)
Partner	260 (8.74)	131 (12.58)	44 (6.59)	435 (7.81)
Separated	286 (7.16)	128 (5.37)	37 (3.11)	451 (5.08)
Divorced	524 (11.75)	178 (9.29)	147 (13.06)	849 (12.31)
Widowed	353 (7.90)	78 (4.29)	103 (7.83)	534 (7.74)
Never married	1170 (31.55)	542 (30.92)	173 (22.05)	1885 (26.81)
Region				
Northeast	411 (15.69)	1135 (55.69)	107 (22.67)	1653 (20.56)
Midwest	595 (18.81)	12 (4.05)	83 (7.96)	690 (12.91)
South	2330 (56.24)	456 (29.11)	609 (54.60)	3395 (54.48)
West	234 (9.25)	18 (11.14)	92 (14.76)	344 (12.06)
	Mean (SD^a^)	Mean (SD)	Mean (SD)	Mean (SD)
Education	12.43 (2.23)	12.93 (1.00)	13.32 (5.00)	12.89 (2.65)
Age (Years)	42.33 (14.50)	40.28 (5.78)	44.98 (31.11)	43.57 (16.61)
Income ($ US)	36,846 (33,236)	47,017 (15,242)	47,397 (75,266)	42,455 (39,594)

^a^ *SD* standard deviation

^b^Weights have been considered

In summary, number of medical comorbidities was positively associated with lifetime MDD among non-Hispanic Whites (OR = 1.719, 95 % CI = 1.018 – 2.902) and African Americans (OR = 1.235, 95 % CI = 1.056– 1.445) but not Caribbean Blacks (*P* > .05) with diabetes (Table 2). Number of medical comorbidities was associated with 12-month MDD among non-Hispanic Whites (OR = 1.757, 95 % CI = 1.119 – 2.759) and African Americans (OR = 1.381, 95 % CI = 1.175 – 1.623) but not Caribbean Blacks (*P* > .05) with diabetes (Table 3).

**Table 2:** Association between number of medical comorbidities and lifetime major depressive disorder among non-Hispanic Whites, African Americans, and Caribbean Blacks with diabetes

	Odds ratio	Linearized SE	t	*P*	95 % CI for odds ratio	
All						
Age	0.917	0.017	−4.8	<0.001	0.885	0.951
Race/Ethnicity^a^						
African American	1.664	1.302	0.65	0.517	0.349	7.931
Non-Latino White	5.285	4.888	1.8	0.076	0.835	33.472
Marital Status^b^						
Divorced/Separated/Widowed	2.697	1.268	2.11	0.039	1.055	6.895
Never Married	0.767	0.518	−0.39	0.695	0.199	2.949
Region^c^						
Midwest	1.094	0.716	0.14	0.891	0.296	4.043
South	0.356	0.188	−1.96	0.055	0.124	1.021
West	0.051	0.060	−2.5	0.015	0.005	0.548
Number of Comorbid Medical Conditions	1.365	0.137	3.11	0.003	1.118	1.668
Non-Hispanic Whites						
Age	0.843	0.039	−3.67	0.003	0.763	0.932
Marital Status						
Divorced/Separated/Widowed	24.457	35.014	2.23	0.042	1.135	527.129
Never Married	137.105	244.211	2.76	0.015	3.006	6254.341
Region						
Midwest	7.292	11.484	1.26	0.228	0.249	213.752
South	0.152	0.199	−1.44	0.172	0.009	2.513
West	0.001	0.003	−3.49	0.004	0.000	0.079
Number of Comorbid Medical Conditions	1.719	0.420	2.22	0.043	1.018	2.902
African Americans						
Age	0.939	0.017	−3.41	0.002	0.904	0.975
Marital Status^b^						
Divorced/Separated/Widowed	2.076	1.007	1.51	0.143	0.770	5.597
Never Married	0.520	0.408	−0.83	0.411	0.105	2.584
Region^c^						
Midwest	0.751	0.571	−0.38	0.709	0.159	3.554
South	0.431	0.225	−1.61	0.118	0.149	1.252
West	1.000					
Number of Comorbid Medical Conditions	1.235	0.095	2.76	0.010	1.056	1.445
Caribbean Blacks						
Age	0.950	0.033	−1.45	0.161	0.883	1.022
Marital Status^b^						
Divorced/Separated/Widowed	2.600	3.130	0.79	0.436	0.214	31.580
Never Married	1.650	2.107	0.39	0.699	0.117	23.315
Region^c^						
Midwest	1.000					
South	1.181	0.992	0.2	0.845	0.207	6.744
West	1.000					
Number of Comorbid Medical Conditions	1.379	0.260	1.7	0.103	0.932	2.041

*SE* Standard error

*CI* Confidence interval

^a^Reference Group = Caribbean Blacks

^b^Reference Group = Married

^c^Reference Group = Northeast

**Table 3:** Association between number of medical comorbidity and Odds of 12-month major depressive disorder among non-Hispanic Whites, African Americans, and Caribbean Blacks with diabetes

	Odds ratio	Linearized SE	t	*P*	95 % CI for odds ratio	
All						
Age	0.912	0.019	<0.001	<0.001	0.875	0.951
Race/Ethnicity^a^						
African American	1.536	1.412	0.47	0.642	0.245	9.626
Non-Latino White	2.078	2.183	0.7	0.489	0.255	16.912
Marital Status^b^						
Divorced/Separated/Widowed	3.802	2.233	2.27	0.026	1.178	12.275
Never Married	2.249	1.577	1.16	0.252	0.554	9.120
Region^c^						
Midwest	0.232	0.220	−1.54	0.128	0.035	1.542
South	0.297	0.178	−2.02	0.047	0.090	0.984
West	0.102	0.090	−2.59	0.012	0.018	0.591
Number of Comorbid Medical Conditions	1.531	0.144	4.54	0.000	1.270	1.847
Non-Hispanic Whites						
Age	0.804	0.058	−3.01	0.010	0.687	0.940
Marital Status^b^						
Divorced/Separated/Widowed	16.478	26.468	1.74	0.105	0.513	529.636
Never Married	252.770	503.945	2.77	0.016	3.405	18761.970
Region^c^						
Midwest	1.000					
South	0.018	0.035	−2.01	0.065	0.000	1.339
West	0.000	0.001	−3.22	0.007	0.000	0.072
Number of Comorbid Medical Conditions	1.757	0.367	2.7	0.018	1.119	2.759
African Americans						
Age	0.917	0.021	−3.82	0.001	0.875	0.960
Marital Status^b^						
Divorced/Separated/Widowed	4.691	3.536	2.05	0.049	1.004	21.913
Never Married	1.329	1.140	0.33	0.743	0.230	7.681
Region^c^						
Midwest	0.434	0.398	−0.91	0.370	0.066	2.833
South	0.480	0.309	−1.14	0.263	0.129	1.789
West	1.000					
Number of Comorbid Medical Conditions	1.381	0.109	4.09	0.000	1.175	1.623
**Caribbean Blacks**						
Age	0.918	0.042	−1.85	0.078	0.834	1.011
Marital Status^b^						
Divorced/Separated/Widowed	1.740	1.853	0.52	0.608	0.191	15.843
Never Married	0.234	0.409	−0.83	0.415	0.006	8.762
Region^c^						
Midwest	1.000					
South	1.442	1.591	0.33	0.743	0.146	14.215
West	1.000					
Number of Comorbid Medical Conditions	1.296	0.324	1.03	0.312	0.771	2.178

*SE* Standard error

*CI* Confidence interval

^a^Reference Group = Caribbean Blacks

^b^Reference Group = Married

^c^Reference Group = Northeast

## Discussion

The present study documented racial and ethnic variations in the association between number of medical comorbidities and MDD among individuals with diabetes. Our findings suggest that number of medical comorbidities is associated with higher odds of lifetime and 12-month MDD among non-Hispanic Whites and African Americans, but not Caribbean Blacks with diabetes. Although there is a well-established literature on the effect of medical comorbidities in diabetes and other index medical conditions [74, 75], very little is known about race and ethnic differences in these links [18, 52, 60].

Our finding regarding the positive association between number of medical comorbidities and MDD among non-Hispanic White and African American individuals with diabetes is in line with the findings from previous studies [51, 76]. There are also studies not showing any effects for comorbidities on patients’ outcomes [77, 78]. We also could not show an association between number of medical comorbidities and MDD among Caribbean Black individuals with diabetes. The remaining question is what psychosocial factors explain race and ethnic differences in the link between medical and mental health.

Some researchers believe that the effect of somatic comorbidity on well-being and health is independent of type of comorbidity, index disease, outcome, and population. Although medical comorbidities worsen a wide range of objective and subjective outcomes [19], our study suggests that these effects may vary across populations. Thus, although health care providers should take a holistic approach to the subjective well-being of patients with any index disease such as diabetes [19], such interventions can also benefit from tailoring based on race and ethnicity.

Medical comorbidities are rules rather than exceptions [19]. As comorbidities influence multiple aspects of subjective health, health care providers should pay special attention to the existing physical and mental comorbidities; such a comprehensive approach may improve physical and mental well-being of patients [51]. Physicians and other health care providers who deliver care to patients with diabetes should evaluate patients for other comorbid medical and mental conditions including MDD, however, the screening and management protocols that are tailored based on race and ethnicity may be superior in efficacy. In all groups, however, regardless of race and ethnicity, early detection and treatment of comorbid conditions should be considered as a universal goal.

Although it is not only the index medical condition but also comorbid conditions that impose risk to the well-being of patients, health care providers have a tendency to exclusively focus on the index disease. Any medical decision for a patient with diabetes should be made while taking into account all medical and psychiatric comorbid conditions. Unfortunately, less has been discussed about the importance of incorporating medical and psychiatric comorbidities in the process of care for racial and ethnic minority patients with chronic medical conditions such as diabetes [79, 80].

Comorbidity affects prognosis of medical conditions, and diabetes is not an exception [81–85]. Primary health care providers and also specialists should always be encouraged to consider all chronic medical and mental comorbidities in the process of decision-makings regarding treatment choices [79, 80, 86]. The current study provides a better understanding of racial and ethnic differences in the effect of comorbid medical conditions on MDD of patients with diabetes, and this information will hopefully help with the medical decision-making related to the care of patients with diabetes [87].

Before any final conclusion, all limitations of the current study should be discussed. Due to the cross sectional nature of our study, causal association is implausible. The results do not suggest whether MDD is a risk factor for multiple comorbid conditions, or comorbid medical conditions cause MDD. The study did not sample U.S. residents who were not able to undergo an interview in English. Type of chronic comorbid conditions was not entered into the analysis, as well. This is particularly important because various chronic medical conditions may have different psychological correlates [88]. In addition, diabetes and also comorbid medical conditions were measured using self-reported data, which is subjected to measurement error (recall bias). Furthermore, the result of our study is generalizable to the community sample of adults with diabetes, not necessarily to a clinical sample of patients with diabetes. Finally, it was also unknown if comorbid conditions such as heart disease or stroke were complications of diabetes or not. Future research should differentiate between medical comorbidities (such as hypertension, which are independent of the diabetes diagnosis) and medical complications associated with diabetes (e.g. micro-vascular or macro-vascular complications) which are secondary to diabetes. Due to the above limitations, the results should be interpreted with caution. More research is needed to better understand race and ethnic differences in the role of medical comorbidities in shaping psychological well-being of patients with diabetes and other conditions [18, 52, 60, 89, 90].

## Conclusion

Our findings suggest that among individuals with diabetes, race and ethnicity moderate the association between number of medical comorbidities and MDD. This information may help a wide range of health care providers such as endocrinologists, internists, psychiatrists and family physicians who are involved in providing health care for individuals with diabetes. Patients with diabetes should be screened for multiple comorbid medical conditions and MDD, regardless of their race and ethnicity, however, multiple comorbid medical conditions and MDD tend to cluster more strongly among non-Hispanic White and African American than Caribbean Black individuals with diabetes. Further research should explore why the link between number of medical comorbidities and MDD is weaker among individuals with diabetes who are from Caribbean Black descent.

## References

[1] International Diabetes Foundation. Global Burden. http://www.idf.org/diabetesatlas/5e/the-global-burden

[2] Danaei G, Finucane MM, Lu Y, Singh GM, Cowan MJ, Paciorek CJ (2011). National, regional, and global trends in fasting plasma glucose and diabetes prevalence since 1980: systematic analysis of health examination surveys and epidemiological studies with 370 country-years and 2.7 million participants. Lancet.

[3] de Groot M, Anderson R, Freedland KE, Clouse RE, Lustman PJ (2001). Association of depression and diabetes complications: a meta-analysis. Psychosom Med.

[4] Mc Donald S, Sharpe L, Blaszczynski A (2014). The psychosocial impact associated with diabetes-related amputation. Diabet Med.

[5] de Grauw WJ, van de Lisdonk EH, Behr RR, van Gerwen WH, van den Hoogen HJ, van Weel C (1999). The impact of type 2 diabetes mellitus on daily functioning. Fam Pract.

[6] Ryerson B, Tierney EF, Thompson TJ, Engelgau MM, Wang J, Gregg EW (2003). Excess physical limitations among adults with diabetes in the US population, 1997–1999. Diabetes Care.

[7] Gregg EW, Beckles GL, Williamson DF, Leveille SG, Langlois JA, Engelgau MM (2000). Diabetes and physical disability among older US adults. Diabetes Care.

[8] Volpato S, Blaum C, Resnick H, Ferrucci L, Fried LP, Guralnick JM (2002). Women’s health and aging study. Diabetes Care.

[9] Coffey JT, Brandle M, Zhou H, Marriott D, Burke R, Tabaei BP (2002). Valuing health-related quality of life in diabetes. Diabetes Care.

[10] Anderson RJ, Freedland KE, Clouse RE, Lustman PJ (2001). The prevalence of comorbid depression in adults with diabetes: a meta-analysis. Diabetes Care.

[11] Feinstein AR (1970). The pre-therapeutic classification of co-morbidity in chronic disease. J Chron Dis.

[12] Roy T, Lloyd CE (2012). Epidemiology of depression and diabetes: a systematic review. J Affect Disord.

[13] Long AN, Dagogo-Jack S (2011). Comorbidities of diabetes and hypertension: mechanisms and approach to target organ protection. J Clin Hypertens (Greenwich).

[14] Huang Y, Wei X, Wu T, Chen R, Guo A (2013). Collaborative care for patients with depression and diabetes mellitus: a systematic review and meta-analysis. BMC Psychiatry.

[15] Hlatky MA (2004). Comorbidity and outcome in patients with coronary artery disease. J Am Coll Cardiol.

[16] Stewart S (2003). Refractory to medical treatment but not to nursing care: can we do more for patients with chronic angina pectoris?. Eur J Cardiovasc Nurs.

[17] Cleves MA, Sanchez N, Draheim M (1997). Evaluation of two competing methods for calculating Charlson’s comorbidity index when analyzing short-term mortality using administrative data. J Clin Epidemiol.

[18] Assari S (2014). Chronic medical conditions and major depressive disorder: differential role of positive religious coping among African americans, Caribbean blacks and Non-Hispanic whites. Int J Prev Med.

[19] Assari S, Moghani Lankarani M, Ahmadi K (2013). Comorbidity influences multiple aspects of well-being of patients with ischemic heart disease. Int Cardiovasc Res J.

[20] Assari S (2014). Cross-country variation in additive effects of socio-economics, health behaviors, and comorbidities on subjective health of patients with diabetes. J Diabetes Metab Disord.

[21] Hollisaaz MT, Aghanassir M, Lorgard-Dezfuli-Nezad M, Assari S, Hafezie R, Ebrahiminia M (2007). Medical comorbidities after renal transplantation. Transplant Proc.

[22] Brett T, Arnold-Reed DE, Popescu A, Soliman B, Bulsara MK, Fine H (2013). Multimorbidity in patients attending 2 Australian primary care practices. Ann Fam Med.

[23] Booth HP, Prevost AT, Gulliford MC (2014). Impact of body mass index on prevalence of multimorbidity in primary care: cohort study. Fam Pract.

[24] Ornstein SM, Nietert PJ, Jenkins RG, Litvin CB (2013). The prevalence of chronic diseases and multimorbidity in primary care practice: a PPRNet report. J Am Board Fam Med.

[25] Stanners MN, Barton CA, Shakib S, Winefield HR (2012). A qualitative investigation of the impact of multimorbidity on GP diagnosis and treatment of depression in Australia. Aging Ment Health.

[26] Diederichs C, Berger K, Bartels DB (2011). The measurement of multiple chronic diseases–a systematic review on existing multimorbidity indices. J Gerontol A Biol Sci Med Sci.

[27] Salisbury C, Johnson L, Purdy S, Valderas JM, Montgomery AA (2011). Epidemiology and impact of multimorbidity in primary care: a retrospective cohort study. Br J Gen Pract.

[28] Wells KB, Golding JM, Burnam MA (1989). Chronic medical conditions in a sample of the general population with anxiety, affective, and substance use disorders. Am J Psychiatry.

[29] Kazemi-Saleh D, Pishgou B, Farrokhi F, Assari S, Fotros A, Naseri H (2008). Gender impact on the correlation between sexuality and marital relation quality in patients with coronary artery disease. J Sex Med.

[30] Khedmat H, Karami GR, Pourfarziani V, Assari S, Rezailashkajani M, Naghizadeh MM (2007). A logistic regression model for predicting health-related quality of life in kidney transplant recipients. Transplant Proc.

[31] Malekahmadi MR, Rahimzadeh S, Dezfuli Nejad ML, Lankarani MM, Einollahi B, Assari S (2011). Importance of socioeconomic, clinical, and psychological factors on health-related quality of life in adolescents after kidney transplant. Exp Clin Transplant.

[32] Noohi S, Khaghani-Zadeh M, Javadipour M, Assari S, Najafi M, Ebrahiminia M (2007). Anxiety and depression are correlated with higher morbidity after kidney transplantation. Transplant Proc.

[33] Azarkeivan A, Hajibeigi B, Alavian SM, Lankarani MM, Assari S (2009). Associates of poor physical and mental health-related quality of life in beta thalassemia-major/intermedia. J Res Med Sci.

[34] Deyo RA, Cherkin DC, Ciol MA (1992). Adapting a clinical comorbidity index for use with ICD-9-CM administrative databases. J Clin Epidemiol.

[35] van den Akker M, Buntinx F, Knottnerus JA (1996). Comorbidity or multimorbidity: what’s in a name? A review of literature. Eur J Gen Pract.

[36] Hudon C, Fortin M, Dubois MF, Almirall J (2008). Comorbidity and glycemia control among patients with type 2 diabetes in primary care. Diabetes Metab Syndr Obes.

[37] Gjeilo KH, Wahba A, Klepstad P, Lydersen S, Stenseth R (2006). Health-related quality of life three years after coronary surgery: a comparison with the general population. Scand Cardiovasc J.

[38] Chan DSK, Chau JPC, Chang AM (2005). Acute coronary syndromes: cardiac rehabilitation programmes and quality of life. J Adv Nurs.

[39] Pirraglia PA, Peterson JC, Russo PW, Charlson ME (2003). Assessment of decline in health-related quality of life among angina-free patients undergoing coronary artery bypass graft surgery. Cardiology.

[40] Sherman AM, Shumaker SA, Kancler C, Zheng B, Reboussin DM, Legault C (2003). Baseline health-related quality of life in postmenopausal women with coronary heart disease: the estrogen replacement and atherosclerosis (ERA) trial. J Women’s Health.

[41] Kennedy MD, Haykowsky M, Daub B, van Lohuizen K, Knapik G, Black B (2003). Effects of a comprehensive cardiac rehabilitation program on quality of life and exercise tolerance in women: a retrospective analysis. Curr Control Trials Cardiovasc Med.

[42] Echteld MA (2003). Modeling predictors of quality of life after coronary angioplasty. Allnals of Behav Med.

[43] Rumsfeld JS, Ho PM, Magid DJ, McCarthy MJ, Shroyer AL, MaWhinney S (2004). Predictors of health-related quality of life after coronary artery bypass surgery. Ann Thorac Surg.

[44] Sundh J, Johansson G, Larsson K, Lindén A, Löfdahl CG, Janson C, et al. Comorbidity and health-related quality of life in patients with severe chronic obstructive pulmonary disease attending Swedish secondary care units. Int J Chron Obstruct Pulmon Dis. 2015;10:173–83. 10.2147/COPD.S74645 PMC4310343 25653516

[45] Chen W, Lynd LD, FitzGerald JM, Marra CA, Rousseau R, Sadatsafavi M. The added effect of comorbidity on health-related quality of life in patients with asthma. Qual Life Res. 2015 Jun 3. [Epub ahead of print] PubMed PMID: 26038225. 10.1007/s11136-015-0995-6 26038225

[46] Sherman AM, Shumaker SA, Kancler C, Zheng B, Reboussin DM, Legault C, et al. Baseline health-related quality of life in postmenopausal women with coronary heart disease: the estrogen replacement and atherosclerosis (ERA) trial. J Womens Health (Larchmt). 2003;12(4):351–62. 10.1089/154099903765448862 12804342

[47] Karlsson I (2000). Sense of coherence: quality of life before and after coronary artery bypass surgery a longitudinal study. J Adv Nurs.

[48] Westin L, Nilstun T, Carlsson R, Erhardt L (2005). Patients with ischemic heart disease: quality of life predicts long-term mortality. Scand Cardiovasc J.

[49] Powell H, Lim LL-Y, Heller RF. Accuracy of administrative data to assess comorbidity in patients with heart disease: an Australian perspective. J Clin Epidemiol. 2001;54:687–93. 10.1016/s0895-4356(00)00364-4 11438409

[50] American Heart Association (2002). 2003 Heart and stroke statistical update.

[51] Baumeister H, Balke K, Ha¨rter M (2005). Psychiatric and somatic comorbidities are negatively associated with quality of life in physically ill patients. J Clin Epidemiol.

[52] Assari S (2014). Additive effects of anxiety and depression on body mass index among blacks: role of ethnicity and gender. Int Cardiovasc Res J.

[53] Assari S (2014). Separate and combined effects of anxiety, depression and problem drinking on subjective health among Black, Hispanic and Non-Hispanic White men. Int J Prev Med.

[54] Assari S (2015). Cross-Country Differences in the Additive Effects of Socioeconomics, Health Behaviors and Medical Comorbidities on Disability among Older Adults with Heart Disease. J Teh Univ Heart Ctr.

[55] Assari S, Lankarani MM, Lankarani RM (2013). Ethnicity modifies the additive effects of anxiety and drug use disorders on suicidal ideation among black adults in the United States. Int J Prev Med.

[56] Assari S (2014). The link between mental health and obesity: role of individual and contextual factors. Int J Prev Med.

[57] Assari S (2015). Ethnic and gender differences in additive effects of socio-economics, psychiatric disorders, and subjective religiosity on suicidal ideation among blacks. Int J Prev Med.

[58] Assari S, Lankarani MM (2014). Race and ethnic differences in associations between cardiovascular diseases, anxiety, and depression in the United States. Int J Travel Med Glob Health.

[59] Assari S, Caldwell CH (2015). Gender and ethnic differences in the association between obesity and depression among black adolescents. J Racial Ethn Health Disparities.

[60] Watkins DC, Assari S, Johnson-Lawrence V (2015). Race and ethnic group differences in comorbid major depressive disorder, generalized anxiety disorder, and chronic medical conditions. J Racial Ethn Health Disparities.

[61] Jackson JS, Neighbors HW, Nesse RM, Trierweiler SJ, Torres M (2004). Methodological innovations in the national survey of American life. Int J Methods in Psych Res.

[62] Jackson JS, Torres M, Caldwell CH, Neighbors HW, Nesse RM, Taylor RJ (2004). The national survey of american life: a study of racial, ethnic, and cultural influences on mental disorders and mental health. Int J Methods in Psych Res.

[63] Heeringa S, Wagner J, Torres M, Duan NH, Adams T, Berglund P (2004). Sample designs and sampling methods for the collaborative psychiatric epidemiology studies (CPES). Int J Methods in Psych Res.

[64] Interuniversity Consortium for Political and Social Research (ICPSR). National Study of American Life (NSAL) sample design. 2013. http://www.icpsr.umich.edu/icpsrweb/CPES/about_cpes/sample_design.jsp

[65] Lampe FC, Walker M, Lennon LT, Whincup PH, Ebrahim S (1999). Validity of a self-reported history of doctor-diagnosed angina. J Clin Epidemiol.

[66] Kessler RC, Andrews G, Mroczek D, Ustun B, Wittchen H (1998). The World Health Organization composite international diagnostic interview short form (CIDI-SF). Int J Methods in Psych Res.

[67] Robins LN, Wing J, Wittchen HU, Helzer JE, Babor TF, Burke J (1988). The composite international diagnostic interview. An epidemiologic instrument suitable for use in conjunction with different diagnostic systems and in different cultures. Arch Gen Psychiatry.

[68] Wittchen HU (1994). Reliability and validity studies of the WHO–Composite International Diagnostic Interview (CIDI): a critical review. J Psychiatr Res.

[69] Kessler RC, Wittchen HU, Abelson JM, McGonagle K, Schwarz N, Kendler KS (1998). Methodological studies of the Composite International Diagnostic Interview (CIDI) in the US national comorbidity survey (NCS). Int J Methods Psychiatr Res.

[70] Kessler RC, Calabrese JR, Farley PA, Gruber MJ, Jewell MA, Katon W (2013). Composite International Diagnostic Interview screening scales for DSM-IV anxiety and mood disorders. Psychol Med.

[71] Assari S, Lankarani MM, Moazen B (2012). Religious beliefs may reduce the negative effect of psychiatric disorders on age of onset of suicidal ideation among blacks in the United States. Int J Prev Med.

[72] Williams DR, Haile R, Gonzalez HM, Neighbors H, Baser R, Jackson JS (2007). The mental health of Black Caribbean immigrants: results from the National Survey of American Life. Am J Public Health.

[73] Shehatah A, Rabie MA, Al-Shahry A (2010). Prevalence and correlates of depressive disorders in elderly with type 2 diabetes in primary health care settings. J Affect Disord.

[74] Chen J, Radford MJ, Wang Y, Krumholz HM (2000). Care and outcomes of elderly patients with acute myocardial infarction by physician specialty: the effects of comorbidity and functional limitations. Am J Med.

[75] Guralink JM (1996). Assessing the impact of comorbidity in the older population. AEP.

[76] Gijsen R, Hoeyymans N, Schellevis FG, Ruwaard D, Satariano WA, van den Bos GA (2001). Causes and consequences of comorbidity: a review. J Clin Epidemiol.

[77] van Manen JG, Bindels PJE, Dekker FW, IJzermans CJ, van der Zee JS, Schadé E (2002). Risk of depression in patients with chronic obstructive pulmonary disease and its determinants. Thorax.

[78] Zwibel, HL Wilner, AN. Comorbidities and quality of life in patients with multiple sclerosis. 60th Annual Meeting of the American Academy of Neurology. http://www.medscape.org/viewarticle/575291.

[79] Fryback DG, Lawrence WF (1997). Dollars may not buy as many QALYs as we think: a problem with defining quality-of-life adjustments. Med Decis Mak.

[80] Harris RA, Nease RF (1997). The importance of patient preferences for comorbidities in cost-effectiveness analyses. J Health Econ.

[81] Bleichrodt H, Crainich D, Eeckhoudt L (2003). The effect of comorbidities on treatment decisions. J Health Econ.

[82] Kaplan MH, Feinstein AR (1974). The importance of classifying initial comorbidity in evaluating the outcome of diabetes mellitus. J Chron Dis.

[83] Greenfield S, Aronow HU, Elashoff RM, Watanabe D (1988). Flaws in mortality data: the hazards of ignoring comorbid disease. JAMA.

[84] Charlson ME, Pompei PP, Ales KL, MacKenzie CR (1987). A new method of classifying prognostic co-morbidity in longitudinal studies; development and validation. J Chron Dis.

[85] Hlatkey MA, Paul SM, Gortner SR (1994). Functional capacity after cardiac surgery in elderly patients. J Am Co11 Cardiol.

[86] Charlson ME, MacKenzie CR, Gold JP, Ales KL, Topkins M, Shires GT (1991). Risk for postoperative congestive heart failure. Surg Gynecol Obstet.

[87] Knaus WA, Wagner DP, Lynn J (1991). Short-term mortality predictions for critically ill hospitalized adults: science and ethics. Science.

[88] Bayat N, Alishiri GH, Salimzadeh A, Izadi M, Saleh DK, Lankarani MM (2011). Symptoms of anxiety and depression: a comparison among patients with different chronic conditions. J Res Med Sci.

[89] Assari S, Lankarani MM (2015). The Association between obesity and weight loss intention weaker among blacks and men than whites and women. J Racial Ethnic Health Disparities.

[90] Assari S, Burgard S, Zivin K (2015). Long-Term Reciprocal Associations Between Depressive Symptoms and Number of Chronic Medical Conditions: Longitudinal Support for Black–White Health Paradox. J Racial Ethnic Health Disparities.

